# Role of microRNAs and their downstream target transcription factors in zebrafish thrombopoiesis

**DOI:** 10.1038/s41598-023-42868-7

**Published:** 2023-09-26

**Authors:** Ayah Al Qaryoute, Weam Fallatah, Sanchi Dhinoja, Revathi Raman, Pudur Jagadeeswaran

**Affiliations:** Department of Biological Sciences, University of North Texas, 1511 West Sycamore Street, Denton, TX 76203 USA

**Keywords:** Genetics, Systems biology, Pathogenesis

## Abstract

Previous studies have shown that human platelets and megakaryocytes carry microRNAs suggesting their role in platelet function and megakaryocyte development, respectively. However, a comprehensive study on the microRNAs and their targets has not been undertaken. Zebrafish thrombocytes could be used as a model to study their role in megakaryocyte maturation and platelet function because thrombocytes have both megakaryocyte features and platelet properties. In our laboratory, we identified 15 microRNAs in thrombocytes using single-cell RNA sequencing. We knocked down each of these 15 microRNAs by the piggyback method and found knockdown of three microRNAs, *mir-7148, let-7b,* and *mir-223* in adult zebrafish led to an increase in the percentage of thrombocytes. Functional thrombocyte analysis using plate tilt assay showed no modulatory effect of the three microRNAs on thrombocyte aggregation/agglutination. We also found enhanced thrombosis using arterial laser thrombosis assay in a group of zebrafish larvae after *mir-7148, let-7b*, and *mir-223* knockdowns. These results suggested *mir-7148, let-7b*, and *mir-223* are repressors for thrombocyte production. We then explored miRWalk database for *let-7b* downstream targets and then selected those that are expressed in thrombocytes, and from this list based on their role in differentiation selected 14 genes, *rorca, tgif1, rfx1a, deaf1, zbtb18, mafba, cebpa, spi1a, spi1b, fhl3b, ikzf1, irf5, irf8*, and *lbx1b* that encode transcriptional regulators. The qRT-PCR analysis of expression levels of the above genes following *let-7b* knockdown showed changes in the expression of 13 targets. We then studied the effect of the 13 targets on thrombocyte production and identified 5 genes, *irf5, tgif1, irf8, cebpa,* and *rorca* that showed thrombocytosis and one gene, *ikzf1* that showed thrombocytopenia. Furthermore, we tested whether *mir-223* regulates any of the above 13 transcription factors after *mir-223* knockdown using qRT-PCR. Six of the 13 genes showed similar gene expression as observed with *let-7b* knockdown and 7 genes showed opposing results. Thus, our results suggested a possible regulatory network in common with both *let-7b* and *mir-223*. We also identified that *tgif1, cebpa, ikzf1, irf5*, *irf8,* and *ikzf1* play a role in thrombopoiesis. Since the *ikzf1* gene showed a differential expression profile in *let-7b* and *mir-223* knockdowns but resulted in thrombocytopenia in *ikzf1* knockdown in both adults and larvae we also studied an *ikzf1* mutant and showed the mutant had thrombocytopenia. Taken together, these studies showed that thrombopoiesis is controlled by a network of transcription regulators that are regulated by multiple microRNAs in both positive and negative manner resulting in overall inhibition of thrombopoiesis.

## Introduction

MicroRNAs are well known to regulate gene expression mainly by degradation of the target mRNA through their interaction at the 3`UTR region resulting in a lack of protein translation^1,2^, although a few studies also suggested translational inhibition precedes mRNA degradation^3,4^. Target prediction studies also have shown that miRNAs can bind to the 5`UTR, gene promoter, and coding sequence of the target gene^5,6^. miRNA interaction with the target gene promotor region has been shown to upregulate gene expression and thus promote more protein synthesis^6^. Both positive and negative transcriptional regulation of miRNA-target genes network has been shown to occur in mammals^7^. Most miRNAs are predicted to target multiple mRNAs, and most mRNAs have predicted binding sites for multiple miRNAs. These predictions were based on different algorithms and are theoretical predictions of the targets, but experimental validation is required to verify the candidate miRNA targets^8^.

Mammalian platelets are anucleate cell fragments that play a critical role in primary hemostasis. They are produced from the nucleated megakaryocyte precursors and inherit a number of diverse RNAs such as messenger RNAs and small non-coding mature and premature miRNAs^9^. The premature microRNAs are processed to produce the mature microRNAs that regulate their target mRNA translation and in turn, regulate platelet function^10,11^. In individuals with essential thrombocythemia (ET), thrombocytopenia, and leukemia, dysregulation of platelet microRNA expression and their levels have been noted suggesting that they may affect platelet counts and function^12,13^. Several studies have shown that platelet microRNAs play an important role in regulating platelet protein processing and expression^13^. Likewise, microRNAs have been shown to be involved in megakaryocyte differentiation.

Since zebrafish have thrombocytes which are functionally equivalent to mammalian platelets and also have features similar to megakaryocytes, it has become a good model to investigate the platelet function and their production^14^. Recently, we performed single-cell RNAseq analysis and identified the RNAs that are expressed in thrombocytes. We found a total of 15 different microRNAs in this database and identified knockdowns of three microRNAs *let-7b*, *mir-223* and *mir-7148* resulted in a significant increase in the percentage of total thrombocytes in adults and a rapid time to occlusion in larvae in an arterial laser injury assay.

Using online miRWalk database, thrombocyte RNAseq data and PANTHER programs, we identified 139 downstream transcription factor targets for *let-7b* and experimentally validated individual expression of each of the selected 14 targets based on their role in cell differentiation after *let-7b* knockdown in zebrafish. We then tested whether these 13 targets are also controlled by *mir-223*. We found 13 of them were regulated by these two microRNAs either in a positive or negative manner. Knockdown of each of these 13 factors on thrombocyte production in the adult zebrafish identified knockdown of *irf5, irf8, cebpa, rorca* and *tgif1* showed thrombocytosis and *ikzf1* knockdown showed thrombocytopenia. In arterial laser thrombosis, however, *irf8* knockdown showed shortened TTO consistent with thrombocytosis and *ikzf1* knockdown showed prolonged TTO consistent with thrombocytopenia. We then analyzed *ikzf1* mutant and showed prolonged TTO indicating thrombocytopenia in heterozygote larvae. Taken together these results provide the novel data on *let-7b* and *mir-223* downstream targets that control thrombocyte production both in a positive and negative manner. In summary, *let-7b* and *mir-223* appear to impede thrombocyte production by regulating a complex network of transcription factors that cumulatively slows down the advancement of thrombopoiesis.

## Materials and methods

### Zebrafish husbandry and breeding

Adult wild-type (WT) zebrafish were purchased from Ekk-Will (Tropical Fish Farm, Gibsonton, FL), and were maintained at 28 °C (82°F) recirculating system of deionized water supplemented with Instant Ocean sea salt at pH 7.6^15^. The zebrafish were under a 14-hour light:10-hour dark cycle and fed with live brine shrimp and fish flakes 3 times a day. The embryos were obtained by breeding adult females and males that were placed in a breeding tank separated by a divider overnight. The following morning, when the lights were turned on, the divider was removed to allow the fish to breed and lay eggs. The fertilized eggs were collected and transferred to embryonic E3 medium (0.17 mM KCl, 5 mM NaCl, 0.33 mM CaCl2, and 0.33 mM MgSO4, pH 7.2, and 0.1% methylene blue) in small clean plastic containers and kept in 28 °C (82 °F) incubator. The hatched larvae were used in subsequent experiments.

### Piggyback knockdown into adult zebrafish

Zebrafish cDNA sequences were obtained using the Ensembl Genome Browser. A specific antisense oligonucleotide sequence of 25 nucleotides was selected for each sequence using Primer 3 software. To each of these sequences, at the 3`-end, a 15-base nucleotide sequence 5’-TATAAATTGTAACTG-3’ that is partially complementary to the control Vivo morpholino (VMO), 5′-CCTCTTACCTCAGTTACAATTTATA-3′ (Gene-Tools LLC, Philomath, OR), was added, and the final antisense oligonucleotide (ASO) was purchased from Invitrogen (Grand Island, NY). Piggyback primers are shown in Table 1. The VMO/ASO hybrid was prepared by mixing 15 μl of 0.5 mM VMO with 15 μl of 0.5 mM of ASO, and 3 μl of hybridization buffer (500 mM NaCl, 10 mM Tris-HCl (pH 8.0), and 1 mM EDTA (pH 8.0). The mixture was heated to 94 °C for 5 minutes and slowly cooled and held at 4 °C using a Takara PCR Thermal Cycler (Takara Bio, Mountain View, CA)^16^. For the adult zebrafish injections, the fish was placed on a dry Kimwipe, the water was gently wiped off using a dry Kimwipe, the head and gills were covered with another wet Kimwipe. Then, using a 27G1¼ gauge needle attached to a 1 ml syringe 5 μl of the above VMO/ASO hybrid was injected intravenously into the inferior vena cava of a zebrafish. The control fish were injected with 5 μl 1XPBS. The injected fish were returned to the tank and at 48 h post-injection, blood was collected and kept on ice for use in further experiments. The study was carried out in compliance with the ARRIVE (Animal Research: Reporting of In Vivo Experiments) guidelines.

**Table 1:** List of primers used for piggyback knockdown.

Gene name	Knockdown primers (5′ to 3′)
*mir-101a*	ACTGTACCTTTAAGATGGACAGCAT
*mir-29b-1*	AAATGGTGCTAGATGCCATAAAA
*mir-451a*	CAGCAGAACCCTTACCATTACTAAA
*mir-7148*	AATTCTTGCAGTATCAGCGAGTATT
*let-7a-5*	ATAGTTATCAGCTGAACAGGGTTTG
*let-7a-6*	ACTACCTCACCTTGAAGAAACACAC
*let-7b*	GGTTGTATAGTTAACTCCTGATGGG
*let-7g-1*	GATCTGGTGTGATCCCAAACTATAC
*mir-144*	TACGATGATATCCTGTCTAGAGAGC
*mir-223*	TCAACCACAGTCTGTCAAATACTCT
*mir-142a*	TGCTTTCTACTTTATGGATGACTGC
*mir-30b*	TAGGATGTTTACAGCGACTACACTG
*mir-16b*	GCAAGTGCTTTGACTCCAATATTTA
*mir-457b*	ATACTGGATAACACCACAATCACCT
*mir-142b*	GCTGTCTACTTTATGAGTGACTGCA
*rorca*	GACACCGCTTTTATGAAGATAGTGT
*lbx1b*	AGGCTACACAGTGGTTTTAAATCAG
*deaf1*	CCAGCTATTGTTGTGTTTGATACAG
*irf5*	TGTAGCTCTTCTTCCTCATCCTCTA
*irf8*	TTAAACTTCCCTTTAAATATTGCCC
*rfx1a*	GGTCTCTTATTCCGAGAGACTTTTC
*tgif1*	TAGGCATTGTATCTGTGTTGGTAGA
*fhl3b*	GCCACACAATTAGAAGAAAACTCAT
*cebpa*	AGCTAACACACATTCCTAACAGGAC
*zbtb18*	CACACCTTGACGATATCATACATGT
*ikzf1*	TAGCACCTTAAATCCTTCTGATGAC
*spi1a*	TGTTTTACTTAAACATGCTGCACAT
*spi1b*	AAATTTCCTCTTACCAAATGAAACC
*mafba*	CTACGAAACGAGGGGTAGTACTACA

### Blood collection from adult zebrafish

The VMO/ASO-injected adult zebrafish were laid on their sides on a dry paper towel, and the head and gills were covered with a wet Kimwipe. The skin surface was gently wiped with a dry Kimwipe to remove the water. Using a sharp straight Noyes Scissors 4.7 (World Precision Instruments, Sarasota, FL), a lateral incision was made at the midpoint between the dorsal and ventral fins into the region of the dorsal aorta and posterior cardinal vein. A micropipette tip was used to rapidly collect 2 µl of blood welling up at the site of incision and immediately added into a 1.5 mL Eppendorf tube containing 0.5 µl of 3.8% sodium citrate. The tube was gently mixed by finger tapping and was kept on ice and used in further experiments. This procedure was approved by the Institutional Animal Care and Use Committee of the University of North Texas, and the animal experiments were performed in compliance with institutional guidelines.

### Flow cytometry to estimate the percentage of total thrombocytes

From 6 individual control 1XPBS- and experimental VMO/ASO hybrid-injected adult zebrafish, 1 µl of blood was collected 48 hours post-injection and added into 1.5 mL Eppendorf tube containing 500 µl of 1XPBS, and the tube was gently finger tapped for uniform mixing. The sample was analyzed for 10,000 events using the BD Accuri™ C6 plus flow cytometer. Gating was used to separate the thrombocytes from other blood cells using the forward (FSC) and side scatter (SSC) to generate a scatter plot and the percentage of total thrombocytes in that gate was calculated^17^.

### Whole blood aggregation/agglutination plate-tilt assay

A microwell plate with conical wells of 10 μl volume was used for this assay. Six adult zebrafish were used for each of the WT control and experimental sets. Two separate wells one for the control sample and the second for the experimental sample were used. To each well 8 μl of 1XPBS was added along with 1 μl of 0.2 mM ADP or 1 mg/ml collagen or 3 mg/ml ristocetin (BIO/DATA Corporation, Horsham, PA) followed by the addition of 0.5 μl of citrated blood from either the control fish or the VMO/ASO-injected fish. After 3 to 5 minutes, the plate was tilted at 45° for 5 s^18^. A photograph of the blood migration down in each well was acquired and zoomed in PowerPoint. The length of blood migration from the origin of the well down the walls was measured in centimeters using the ruler in PowerPoint and plotted. The lack of blood migration or stasis is indicative of a positive aggregation/agglutination in response to any of the three agonists^18^.

### In vivo piggyback hybrid injections into zebrafish larvae

A glass capillary of 3 inches length, OD 1.0 mm, with no filament (World Precision Instruments, Sarasota, Florida, USA) was pulled by a vertical pipette puller (David Kopf Instruments, Tujunga, California, USA). The tip was then clipped using a sharp straight Noyes Scissors 4.7 (World Precision Instruments, Sarasota, FL), and loaded with 5 µl of the VMO/ASO-hybrid prepared as described above. Three days post fertilization (dpf) larvae were anesthetized by transferring them using a plastic transfer pipette into a 0.64 mM Tricaine (pH 7.0) and incubating it for 5 seconds. The anesthetized larvae were laid on a 1.2% agarose plate and injected intravenously into the common cardinal vein with 15 nl of VMO/ASO-hybrid using Picospritzer III (Parker Precision Fluidics, Hollis, NH) and a micromanipulator under a dissection microscope. Following the injection, the larvae were transferred to E3 embryonic medium in a plastic cup at 28 °C and incubated for 48 hours for use in laser-induced arterial thrombosis assay.

### Arterial laser thrombosis assay

For laser-induced arterial thrombosis, 3 dpf zebrafish larvae were transferred using a transfer pipette into a 1.5 mL Eppendorf tube containing 0.5 ml E3 medium, and 10 µl of 10 mM Tricaine was added to anesthetize the larvae. After 3 minutes (once the larvae were anesthetized), 0.5 mL of 1.6% low-melting agarose at 37 °C was added. Using a transfer pipette, the contents were mixed gently by pipetting up and down and the larvae along with the contents were then placed into a chamber prepared using a rectangular rubber gasket by pressing it onto a thin coat of petroleum jelly on a microscopic slide^19^. For optimum vessel visibility, the larvae were adjusted with a thin pipette tip such that they were lying on their lateral sides. The slide with the larvae was then placed under a Nikon Optiphot fluorescence microscope and was focused with a 20× objective lens. A pulsed nitrogen laser with a wavelength of 445 nm, routed through the coumarin-440 dye (Micro Point Laser, Stanford Research Systems Inc., Sunnyvale, CA), was delivered at 15 hits per cycle through the fluorescence port such that it hits the caudal artery in the middle of the 5^th^–6th somite region posterior to the anal pore. The time to occlusion (TTO) of the artery was recorded in seconds^19,20^.

### RNA extraction

Adult zebrafish were transferred into a Petri dish containing 40 ml of 1 mM Tricaine in an E3 medium (PH 7.0). Once the anesthetized zebrafish tilted on its side, it was transferred via forceps onto a dry Kimwipe and wiped. Holding the fish in hand, an incision using a pair of sharp straight Noyes Scissors 4.7 (World Precision Instruments, Sarasota, Florida), was made in the ventral surface of the skin from the anal pore up to the gills. The visible liver and spleen were gently extracted using a pair of forceps. To this liver and spleen, 200 µl of TRI Reagent® (Millipore-Sigma, St. Louis, Missouri) was added and the sample was homogenized using the PRO200 MULTI-GEN 7XL Homogenizer (PRO Scientific Inc., Oxford, Connecticut) for 30 seconds^21^. The homogenate was incubated for 10 minutes at room temperature, 20 µl of 1-bromo-3-chloropropane was added and centrifuged for 15 minutes at 10000 × g at 4 °C. The aqueous phase was then transferred into an Eppendorf tube, 200 µl of isopropanol was added, and after 10 minutes, the sample was centrifuged again. The pellet was finally washed at 4 °C with 75% ethanol and suspended in nuclease-free water. The RNA of the 6 individual WT and 6 knockdown samples were pooled into two separate Eppendorf tubes. The pooled total RNA concentration from the WT and knockdown samples was measured using NanoDrop (BioTek, Santa Clara, CA) and then stored at −80 °C for use in further experiments.

### Real-time quantitative reverse transcription PCR

To 1 µl of the RNA (1 µg/µl), 4 µl of qScript cDNA SuperMix (Quanta Bio, Beverly, MA), and 15 µl of nuclease-free water, were added and mixed. The mixture was then incubated using a Takara PCR Thermal Cycler (Takara Bio, Mountain View, CA) for 5 min at 25 °C, followed by 40 min at 42 °C, then 5 min at 85 °C, and finally held at 4 °C. Subsequently, 4 replicates of WT and 4 knockdown samples containing 1 µl of the above cDNA sample was amplified with 5 µl of PowerUp SYBR Green Master Mix (Thermo Fisher Scientific, Grand Island, NY), 3.6 µl of nuclease-free water, and 0.2 µl of each 25 μM gene-specific primers and β-actin primers, listed in Table 2. Quantitative RT-PCR (qRT-PCR) was performed using this mixture for 45 cycles (Chai Open qPCR by Chai, Santa Clara, CA). The data was collected and analyzed for Ct values and fold change.

**Table 2:** List of the forward (FP) and reverse (RP) primers used in qRT-PCR.

Gene name	qRT-PCR primers (5′ to 3′)
*β-actin*	FP: TCTCTTGCTCCTTCCACCAT
	RP: CATCGTACTCCTGCTTGCTG
*mir-223*	FP: CTCTCCTCCTGATCTAGACT
	RP: CCTCTCTTGGGGTATTTGAC
*let-7b*	FP: TCGGACAGGGTGAGGTAGTA
	RP: CCCTTCAGGGAAGGCAGTAG
*mir-7148*	FP: GCTAATACTAGTAATGGAAA
	RP: ACCAATACTAGTATCGGAAA
*rorca*	FP: GACAAGTCAATACAGCACAGAAGA
	RP: GAAGGATAATCTGGTCATTTTGAGA
*lbx1b*	FP: TCTCCATCAAAAATACTTGTCACCT
	RP: GTTTATCCAGAGGGACTAAACCTGT
*deaf1*	FP: CATGATAAAGACGTCTCGAAGTACA
	RP: CCAGCTATTGTTGTGTTTGATACAG
*irf5*	FP: CTTAGTGCCATCGTAGTTGAGTCTG
	RP: CTTAGTGCCATCGTAGTTGAGTCTG
*zbtb18*	FP: AGACATTTGCTACAGTGTCTGAGTG
	RP: CTTTTGTCTAGCTGGTCCTTGTAAA
*rfx1a*	FP: GAGATTCAGAGCTCACCTACACAGT
	RP: CTGAGATGTGGCTTGAACTACTGAC
*tgif1*	FP: GAAGAGGAAGAGGAGAGGAAACTTG
	RP: ATAAACCAGTTGCAAACCTGTAGTG
*fhl3b*	FP: TGCAATGAGTTTTCTTCTAATTGTG
	RP: AGTAGTACTCGTCCTTATCGGGAAT
*irf8*	FP: AATACATGGGCATCTTGAGAAGTAG
	RP: CCTCCGTAGTAGAAAGAAATCAACA
*cebpa*	FP: AATAATCACTTGTCAGTCGTTCACA
	RP: GCTGTCTCGTTTAAGGATAATTTGA
*ikzf1*	FP: GAATCGTCTGTCAGAGCTATCTTTC
	RP: TTTGTGACTTGTGCAGGTTGTATAG
*spi1a*	FP: CCCATATACGACTTCTACCCGTATC
	RP: TGGGAGATGTAAGTATACTGTGGAG
*spi1b*	FP: TGTCAGATGAGGAGTGTATGAGAGA
	RP: CATCTTCTGGTAGGTCATCTTTTTG
*mafba*	FP: GAGACAGCTCCCAGTACTCGTATTA
	RP: CAAGCAACTAATCTAATTCCCAGAA

### Prediction of *let-7b* downstream mRNA targets

All *let-7b* miRNA-mRNA targets in zebrafish were obtained from the online miRWalk database version 2 (http://mirwalk.umm.uni-heidelberg.de/)^22^. From the miRWalk website, fish was selected as the species and *let-7b* was entered as the microRNA name then all the targets were exported into an Excel sheet. These targets were compared with the thrombocyte transcripts from RNAseq analysis, and the thrombocyte-specific targets were selected. Using the Protein Analysis Through Evolutionary Relationships (PANTHER) online software classification database version 16.0 (http://www.pantherdb.org/), all *let-7b* mRNA targets obtained from the miRWalk database were entered in the ID section. Danio rerio was selected as the organism and functional classification analysis was selected. Protein class was selected as the ontology and only targets that are gene-specific transcriptional regulators were obtained^23^.

### *ikzf1* mutant rearing and breeding

From the Zebrafish International Resource Center (ZIRC) *ikzf1*-*sa11269* mutant was obtained as 3 dpf larvae. They were immediately transferred into small clean plastic containers containing embryonic E3 medium and kept at 28 °C (82 °F). At 4 dpf the larvae were fed with live paramecium thrice and the E3 medium was changed daily. The larvae were fed both live paramecium and live brine shrimp twice daily beginning at 9 dpf and until 21 dpf. The E3 medium was gradually replaced with clean system water. At 21 dpf, they were transferred into a recirculating system of deionized water supplemented with Instant Ocean sea salt at pH 7.61 and maintained at 28 °C (82 °F)^15^. The fish were kept under a 14:10 hours light: dark cycle and fed with live brine shrimp until they reached adulthood. Adult fish were fed with live brine shrimp and fish fakes 3 times per day. To obtaining larvae for laser-induced arterial thrombosis, one *ikzf1* heterozygous *ikzf1*^*+/−*^ was crossed with WT *ikzf1*^*+/+*^ from the same progeny as described above.

### DNA extraction from adult zebrafish

For isolating genomic DNA, the zebrafish tail was clipped using sharp straight Noyes Scissors 4.7 (World Precision Instruments, Sarasota, FL). The tail clip from each fish was placed in a 1.5 mL Eppendorf tube containing 50 µL DNA extraction buffer (50 mM KCl, 10 mM Tris, HCl pH 8.5, 0.01% gelatin, 0.45% NP-40, 0.45% Tween20, and 5 mM EDTA) and 0.5 µL (20 mg/mL) of proteinase K (NEB, Ipswich, MA) was added and incubated overnight at 55 °C. The samples were then transferred into a 96 °C water bath and incubated for 10 minutes to inactivate proteinase K. Samples were then centrifuged at 3000 rpm at room temperature (RT) and the supernatant containing genomic DNA was transferred to 1.5 mL Eppendorf tube and stored at −20 °C to be used in mutant screening and genotyping.

### Mutant screening and sequencing

Four µL of the DNA was amplified by PCR using the 10 µL 1-Drop PCR Mix (101Bio, Palo Alto, CA) with the forward primer 5′- CGCAGATGTCCAGTGAGAGC-3 and reverse primer 5`- GGTGAAAGGCCATTCCAGTG-3`. The amplified DNA was resolved by 1.2% agarose gel electrophoresis and the amplified band of size 158 bp was then excised using a scalpel blade and transferred into a 1.5 mL Eppendorf tube and purified using EZNA gel electrophoresis kit (Omega BioTek, Norcross, GA). The purified DNA sample was then sent for Sanger’s Sequencing to GENEWIZ (South Plainfield, NJ). The chromatograms were further analyzed to determine the genotype of the fish using the software FINCH TV 1.5.0 (Geospiza, Inc., Seattle, WA).

### Bioinformatics

Zebrafish gene and the cDNA sequences were retrieved from Ensembl genome browser. The predicted translational products were compared with the human sequences using the publicly available MultAlin program.

### Statistical analysis

Statistical analysis of experimental data was performed using GraphPad Prism version 9.4.1 software (GraphPad Software, San Diego, California, USA). Control and experimental groups were analyzed by student’s *t* test or one-way ANOVA followed by Dunnett’s post-hoc multiple comparison test. *p* value <0.05 was considered significant. *, **, ***, **** and ns (non-significant) represent *p*≤0.05, *p*≤0.01, *p*≤0.001, *p*≤0.0001 and *p*>0.05, respectively. Error bars represent standard deviation. In cases where the experimental groups exhibited a non-normal distribution, we employed Levene's test to assess the assumption of homogeneity of variance between the control and experimental groups. If Levene's test produced a statistically significant result, it would indicate a deviation from the assumption of equal variances. Consequently, we employed Welch's *t* test to compare the means of the control and experimental groups. A *p* value of less than 0.05 would denote a statistically significant distinction between the WT and KD groups.

## Results

### Piggyback hybrid knockdowns of microRNAs in adult zebrafish and estimation of total thrombocyte counts

**Figure 1:**
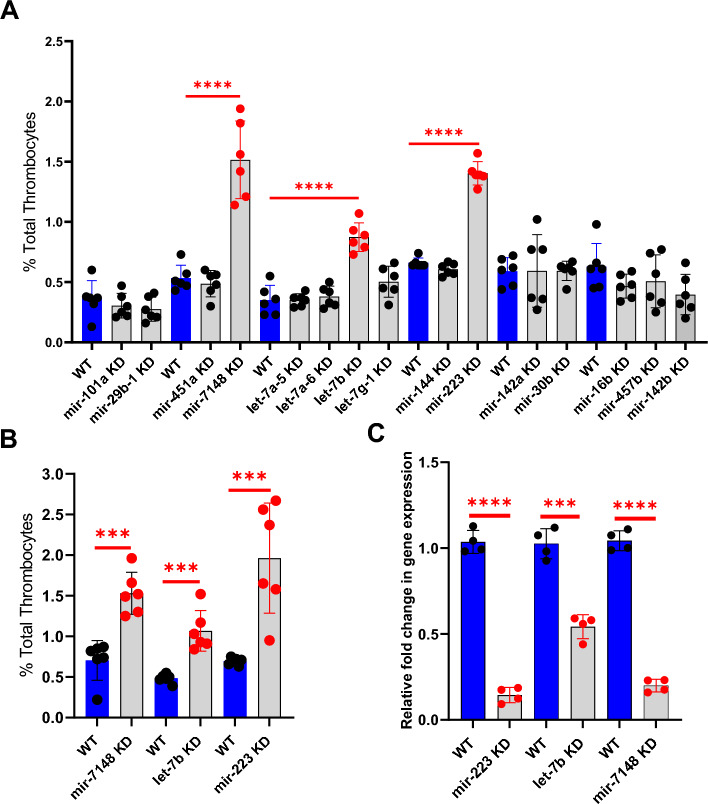
Knockdown screen of microRNAs and thrombocyte production. (**A**) Knockdown screen of the fifteen-microRNAs and estimation of percentage of zebrafish thrombocytes in whole blood compared to daily wild-type (WT). (**B**) Confirmation of the results obtained from knockdown screen on the three microRNAs, *mir-7148, let-7b*, and *mir-223* compared to daily WT injected with 1XPBS (WT). (**C**) Levels of *mir-223*, *let-7b*, and *mir-7148* microRNA. Quantitative real-time PCR showing the fold change of *mir-223, let-7b,* and *mir-7148* gene expression in liver and spleen after their knockdown (KD). The blue bars represent daily WT controls. All three knockdowns are shown as grey bars to the right of each WT daily controls. Error bars represent mean ± SD. Four replicate RNA samples pooled from six fish were used for each of *mir-223*, *let-7b*, and *mir-7148* gene knockdowns, and control experiments (N = 4). The lines on the top of the bars represent a significant difference in fold change in gene expression between WT and knockdown samples. For (**A**) and (**B**), The blue bars represent daily WT controls injected with 1XPBS. Knockdowns of microRNAs are shown as grey bars to the right of each daily WT control. Six fish were used for each of the microRNA-knockdowns as well as control experiments (N = 6). The lines on the top of the bars represent a significant difference in the percentage of thrombocytes between WT and knockdown samples. For (**A**), (**B**) and (**C**) error bars represent mean ± SD. *P* value < 0.05 was considered significant. *** and **** represent *p* ≤ 0.001 and *p* ≤ 0.0001, respectively.

Previous work from our laboratory has identified genes expressed in zebrafish thrombocytes through RNASeq analysis by 10X genomic sequencing to identify the genes involved in thrombocyte differentiation and function^24^. A total of fifteen miRNA genes were found among these expressed genes (*mir-101a, mir-29b-1, mir-451a, mir-7148, let-7a-5, let-7ba-6, let-7b, let-7g-1, mir-144, mir-223, mir-142a, mir-30b, mir-16b, mir-457b,* and *mir-142b*). To investigate the role of these microRNAs in zebrafish thrombocyte production, we used the piggyback knockdown approach to knockdown all the above 15 miRNAs. We prepared individual VMO/ASO sequences for each of the 15 microRNAs. We then used the piggyback knockdown method that was established in our laboratory and injected each of these 15 VMO/ASO hybrids into six fishes. Forty-eight hours after injections, the number of thrombocytes in whole blood was counted using flow cytometry, and their percentages were calculated^17^. For daily controls 1xPBS was injected. This knockdown screen showed that *mir-7148, let-7b,* and *mir-223* knockdowns resulted in a significant increase in the percentage of total thrombocytes (thrombocytosis) (Figure 1A). We confirmed these results by repeating the knockdowns of *mir-7148, let-7b*, and *mir-223* and measuring the thrombocyte percentages. The results are shown in Figure 1B.

### Demonstration of microRNA knockdown through real-time quantitative reverse transcription PCR

Since thrombocytes were counted in the fish blood, to use the same fish for qRT-PCR is not possible because the fish blood collection is terminal. Therefore, we retrieved liver and spleen from the fish that were used for collecting blood since thrombocytes are also found in vascularized liver and spleen. We extracted RNA from the liver and spleen of the individual *mir-7148, let-7b,* and *mir-223* knockdown zebrafish to check whether knockdown occurs. This RNA was subjected to qRT-PCR using *mir-7148, let-7b*, and *mir-233* qRT-PCR primers, respectively. We used β-actin primers as an internal control. The results showed fold change in the gene expression of the VMO/ASO-injected fish with each of the three microRNAs *mir-7148, let-7b*, and *mir-233* was significantly reduced supporting the knockdown occurs at 50–85% levels (Figure 1C). We conducted a similar quantification analysis for the remaining 12 miRNAs, and our results revealed a significant decrease in the levels of these miRNAs following the knockdown experiments (Figure S1). The specific primers utilized for the qRT-PCR assays are shown in supplementary Table S1.

### Plate-tilt assay to study the role of microRNAs on thrombocyte function through whole blood aggregation/agglutination

**Figure 2:**
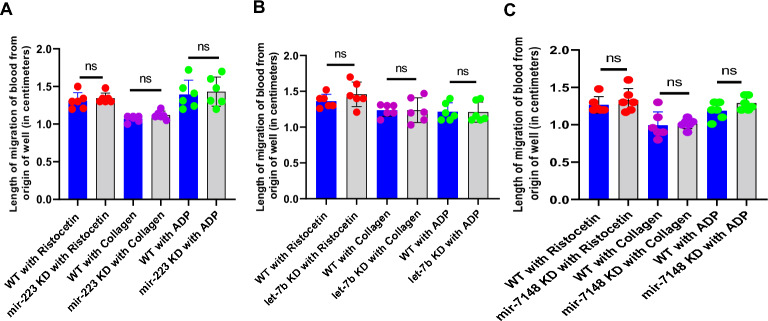
Effect of (**A**) *mir-223*, (**B**) *let-7b*, and (**C**) *mir-7148* knockdowns in adult zebrafish on thrombocyte aggregation/agglutination. Zebrafish blood from the daily controls (WT, represented as blue bars) and the knockdown (KD, represented as grey bars) samples were subjected to whole blood aggregation/agglutination assay with ristocetin, collagen and ADP. The length of migration of blood from the origin of the well down the wall was measured and plotted. The results between WT and KD samples were compared. Six fish were used for each of the knockdown and control experiments (N = 6). The lines on the top of the bars represent a significant difference between WT and knockdown sample sets. Error bars represent mean ± SD. ns (non-significant) represents *p*>0.05.

To study the role of *mir-223, let-7b*, and *mir-7148* on thrombocyte function, we used the plate tilt assay to identify whether there are any differences in thrombocyte aggregation/agglutination in response to the three major platelet agonists, ristocetin, collagen, and ADP after knocking down these microRNAs^18^. We quantified the length of migration of blood from the origin of the well down the walls in centimeters and found that there was no significant difference in the migration of blood between the control and *mir-223, let-7b*, and *mir-7148* knockdown experimental samples, indicating that these microRNAs do not affect the role of thrombocyte function and aggregation/agglutination (Figure 2A,B,C). We also performed knockdown experiments targeting these specific microRNAs to assess the impact of the remaining 12 microRNAs on thrombocyte function. Subsequently, we measured thrombocyte function using the above methodology. Our findings indicate that the knockdown of the above microRNAs did not yield any discernible effects on thrombocyte function (Figure S2).

### Laser-induced arterial thrombosis in zebrafish *mir-223, let-7b*, and *mir-7148* knockdown larvae

**Figure 3:**
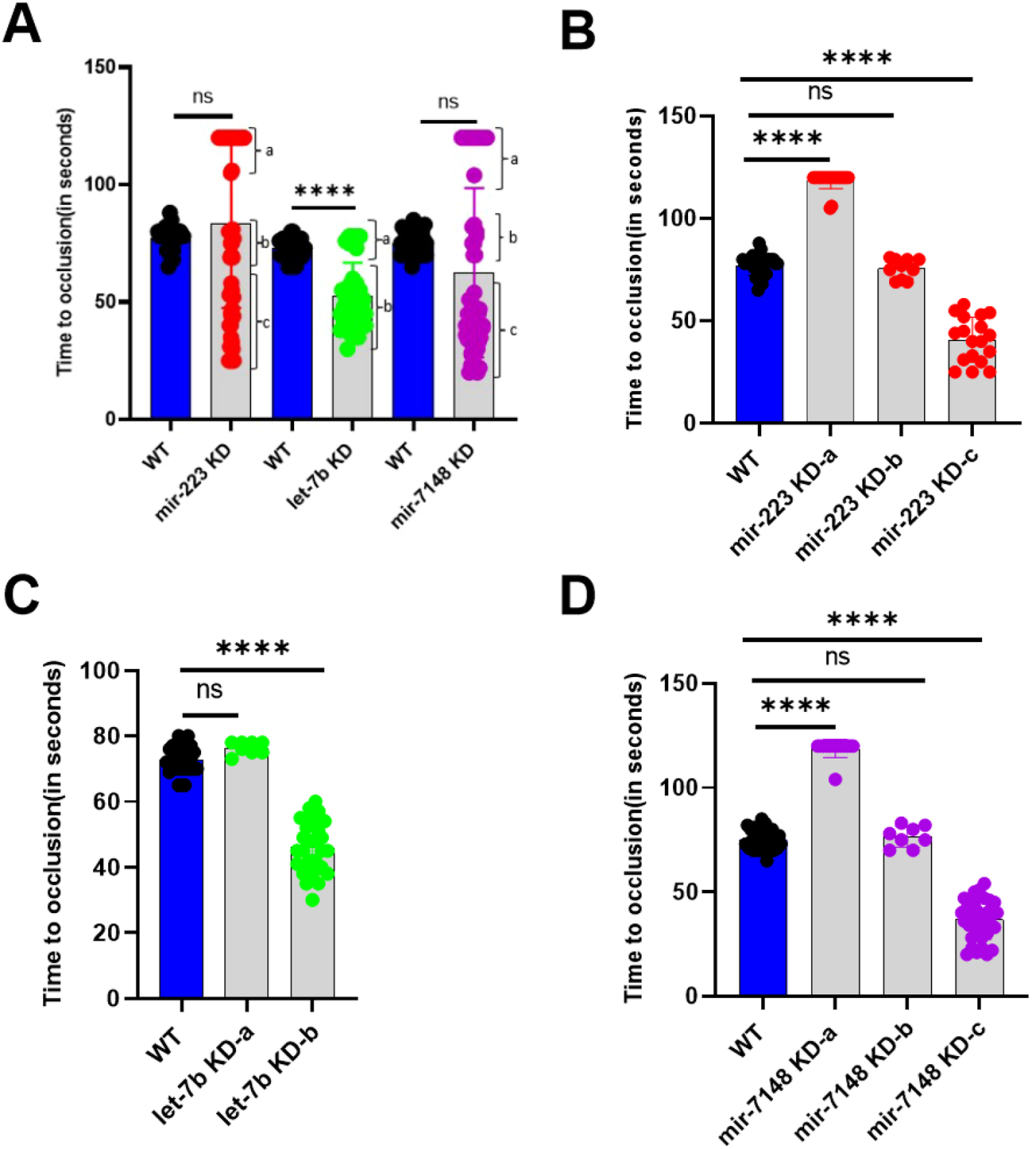
Arterial thrombosis in *mir-223*, *let-7b*, and *mir-7148* knockdown larvae. (**A**) Comparison of time to occlusion of the caudal artery after laser injury between daily WT (blue bars) and knockdown (grey bars) 5 dpf larvae. Knockdown (KD) results of *mir-223*, *let-7b*, and *mir-7148* are shown. The number of larvae used were 30 for daily control WT larvae and 52, 38, and 57 for *mir-223*, *let-7b*, and *mir-7148* KD sets, respectively. Students’ *t* test was used for statistical analysis. Error bars represent mean ± SD. *p* value < 0.05 was considered significant. The lines on the top represent a significant difference between WT and knockdown samples. **** represents *p* ≤ 0.0001. The parentheses show groups a, b and c that show three distinct times to occlusion. (**B**), (**C**) and (**D**) represent comparison of times to occlusion for distinct groups a, b and c obtained from (**A**) for *mir-223*, *let-7b*, and *mir-7148* knockdowns, respectively. In the one-way ANOVA analysis larval numbers obtained from (**A**) for WT were 30. For *mir-223* knockdown 18, 10 and 24 larvae for groups a, b and c, respectively were included. For *let-7b* knockdown 8, and 30 larvae for groups a and b, respectively were chosen. For *mir-7148* knockdown 35, 8 and 14 larvae for groups a, b and c, respectively were included.

To investigate the effects of *mir-223*, *let-7b*, and *mir-7148* knockdowns in zebrafish larvae, we intravenously microinjected the VMO/ASO piggyback hybrid for each of the above 3 microRNAs individually into 3 dpf larvae. On the 5th dpf, the injected larvae were subjected to the laser-induced arterial thrombosis assay to analyze their hemostasis function. We found that the larvae with *let-7b* knockdowns showed an average TTO of 52 seconds that is significantly shorter than the average TTO of daily control larvae of 72 seconds whereas *mir-223*, and *mir-7148* knockdowns showed no significant difference in TTO when compared to control samples (Figure 3A). However, *let-7b* knockdown showed two distinct groups of larvae, group a that had a mean TTO of 76 seconds and group b that had a mean TTO of 45 seconds (Figure 3A,C). Likewise, the *mir-223*, and *mir-7148* knockdown larvae although they did not show a significant difference in TTO compared to WT larvae, they showed three distinct groups, groups a, b and c that had a mean TTO of 118, 75, and 40 seconds for *mir-223* and 118, 76, and 36 seconds, respectively (Figure 3A, Figure 3B,D). When we compared these groups with the respective controls, one of the groups group b in Figure 3B,D and group a in Figure 3C did not show any significant difference. Other groups showed either a significant prolongation of TTO (group a in Figure 3B and Figure 3D) or shortening of TTO (group c in Figure 3B,D and group b in Figure 3C). Thus, in all three microRNA knockdowns we found one group (group c in Figure 3B,D and group b in Figure 3C) that had a mean TTO of 36–40 seconds that is shorter than the daily controls (Figure 3A).

Although considering the inheritance, the above grouping makes sense, since it is not a normal distribution, we used Levene's test to evaluate the homogeneity of variance assumption between WT and *let-7b* knockdown. We obtained a significant result (*p*<0.005) from Levene's test, indicating a violation of the homogeneity of variance assumption. Therefore, Welch's *t* test was applied to compare the means of the two groups. The results of Welch's test revealed a significant difference between WT and *let-7b* knockdown (*p* < 0.001). Likewise, we also obtained significant results with the *miR-7148*. However, Welch's t-test did not yield a significant difference between WT and *miR-223*, although Levene's test showed a *p*<0.005.

To investigate the potential impact of the remaining 12 microRNAs on arterial TTO, we performed knockdown experiments for each of these microRNAs and subsequently assessed TTO using the methodology outlined earlier. Our observations revealed that the knockdown of these 12 microRNAs did not lead to any significant alterations in arterial TTO when compared to the WT control group (Figure S3).

### Selection of downstream target genes for *let-7b* using miRWalk database, RNAseq data and PANTHER classification

First we obtained all *let-7b* targets from miRWalk database and there were 16,488 targets in total. We then compared these targets to all the unique thrombocyte transcripts obtained from the RNAseq analysis and identified 1821 *let-7b* downstream transcripts (attached as an excel file). We then classified these 1821 targets through PANTHER classification system based on the protein class and obtained all 139 gene-specific transcriptional regulator targets. From this, we selected 14 genes *rorca*, *tgif1, rfx1a, deaf1, zbtb18, mafba, cebpa, spi1a, spi1b, ikzf1, irf5, irf8, fhl3b, and lbx1b*. These 14 genes were selected because they have been shown to participate in cell differentiation and hematopoiesis.

### Piggyback knockdown of *let-7b* and quantitative real-time PCR of the 14 transcription factor mRNAs

**Figure 4:**
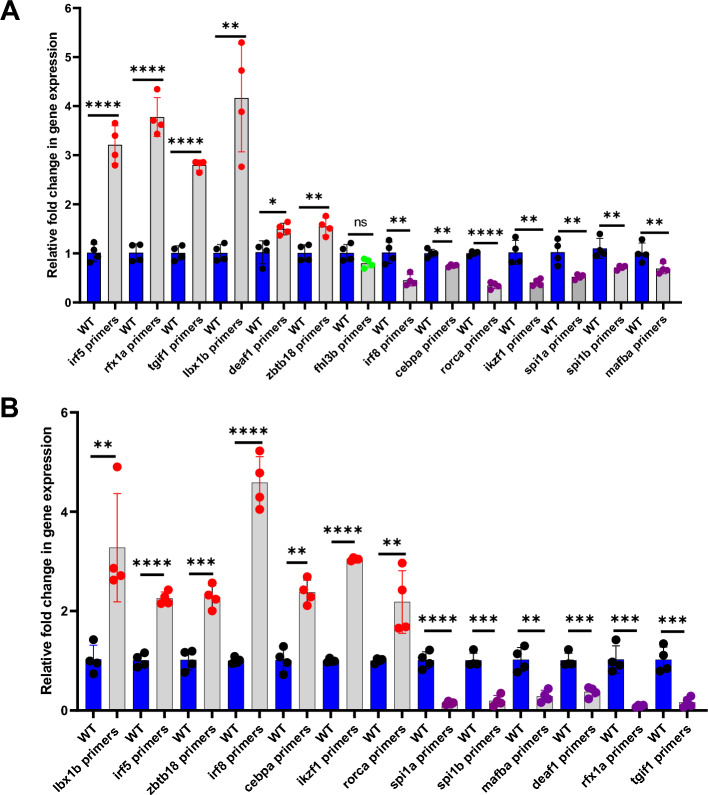
Levels of the downstream transcription factor mRNAs after knockdowns of (**A**) *let-7b* and (**B**) *mir-223*. Quantitative real-time PCR showing the fold change of the transcriptional factor gene expression in daily wild-type (WT) controls (blue bars) and knockdown (KD) samples (grey bars). Four replicates containing six injected fish were used for each replicate sample and control experiments (N = 4). The lines on the top of the bars represent a significant difference between WT and knockdown samples. Error bars represent mean ± SD. *, **, ***, **** and ns (non-significant) represent *p* ≤ 0.05, *p* ≤ 0.01, *p* ≤ 0.001, *p* ≤ 0.0001, *p* >0.05 respectively.

To investigate whether *let-7b* regulates the expression of any of the 14 downstream transcriptional factor genes, we intravenously injected *let-7b* VMO/ASO-hybrid*,* and 48 hours post injection we extracted the RNA from the liver and spleen. This RNA was subjected to qRT-PCR using the forward and reverse primers of *let-7b*. The results showed significant reduction of *let-7b* gene expression confirming the knockdown of *let-7b* in the extracted RNA sample. This RNA was then subjected to qRT-PCR using the forward and reverse primers for each of the 14 transcription factor genes. β-actin was used as an internal control. We identified knockdown of *let-7b* showed a significant increase in *irf5, tgif1, rfx1a, lbx1b, zbtb18,* and *deaf1* transcript levels, a significant decrease in *irf8, cebpa, ikzf1, rorca, mafba, spi1a,* and *spi1b* transcript levels, and no change in *fhl3b* transcript levels (Figure 4A).

### Knockdowns of *let-7b* target genes and analysis of thrombocyte counts

**Figure 5:**
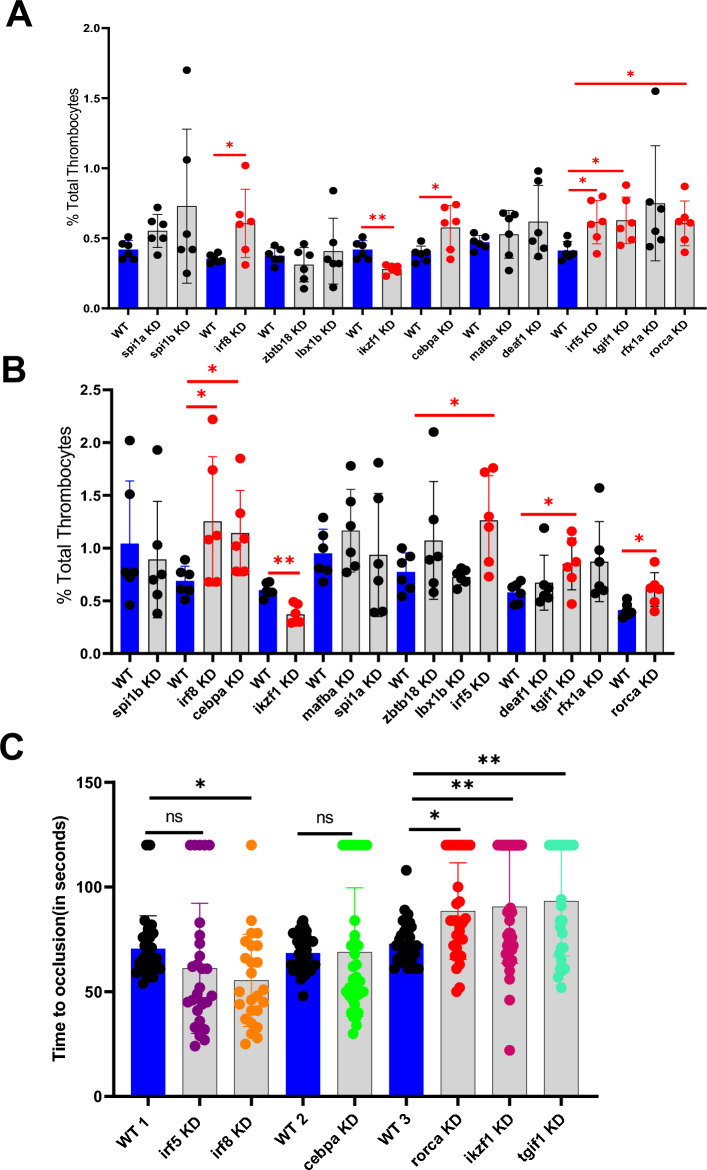
Flow cytometric analysis of total thrombocytes percentage in zebrafish whole blood. (**A**) Knockdown screen of the 13 transcription factors in adult zebrafish compared to daily wild-type (WT). (**B**) Confirmation of the results in (**A**). (**A**) and (**B**), compare the percentage of thrombocytes in whole blood from daily WT controls (blue bars), and knockdown (KD) samples (grey bars). Six fish were used for each of the transcriptional factors and control experiments (N = 6). The red lines on the top of the bars represent a significant difference between WT and knockdown sample sets. Error bars represent mean ± SD. * and ** represent *p* ≤ 0.05, and *p* ≤ 0.01 respectively. *p* value < 0.05 was considered significant. (**C**) Comparison of time to occlusion of the caudal artery after laser injury between 5 dpf daily WT control and knockdown (KD) larvae. The number of larvae used were 30, 29, 25, 40, 35, 31, 31, 42, and 27 in WT 1, *irf5* KD, *irf8* KD, WT 2, *cebpa* KD, WT 3, *rorca* KD, *ikzf1* KD, and *tgif1* KD sets, respectively. The lines on the top represent a significant difference between WT and knockdown samples. Error bars represent mean ± SD. *, **, ***, **** and ns (non-significant) represent *p* ≤ 0.05, *p* ≤ 0.01, *p* ≤ 0.001, *p* ≤ 0.0001, *p* >0.05 respectively.

To study the effect of each of the 14 transcription factors on thrombocyte production in adult zebrafish, we knocked down each of these factors and checked for the percentage of total thrombocytes in the whole blood. We identified that *irf5, tgif1, irf8, cebpa,* and *rorca* knockdowns resulted in a significant increase of the percentage of total thrombocytes; whereas knockdown of *ikzf1* resulted in a significant decrease in the percentage of total thrombocytes; we confirmed the results by repeating this experiment (Figure 5A,B).

### Laser-induced arterial thrombosis in zebrafish *irf5, tgif1, irf8, cebpa, rorca* and *ikzf1* knockdown larvae

To study the role of *irf5, tgif1, irf8, cebpa, rorca* and *ikzf1* on thrombocyte production in zebrafish larvae, we injected the VMO/ASO hybrids for each of the above genes in 3 dpf larvae followed by the laser-induced arterial thrombosis assay in 5 dpf larvae and TTO was measured. *irf8* knockdowns showed a significant shortening of TTO compared to WT larvae. The TTO of *irf5* and *cebpa* knockdown showed no significant change; however, TTOs for *irf5* knockdown showed three groups as seen in *mir-223* and *mir-7148* knockdowns (Figure 5C and Figure 3B and Figure 3D). The TTO in *rorca, ikzf1* and *tgif1* knockdowns showed significant prolongation (Figure 5C). Interestingly, *irf5* and *irf8* knockdowns resulted in death of 25% and 37% larvae, respectively. We also observed microthrombi in 16% and 16.7% of the 5dpf piggyback reagent-injected larvae in *rorca* and *tgif1* knockdowns, respectively (Figure S4 A,B).

### Piggyback knockdown of *mir-223 *and quantitative real-time PCR of the transcription factor target mRNAs controlled by *let-7b*

Since more than one microRNA can regulate a specific mRNA, we investigated whether *mir-223* regulates the 13 transcription factors that are regulated by *let-7b*. We intravenously injected the VMO/ASO hybrid of *mir-223*, and 48 hours post injection we extracted the RNA from the liver and spleen. This RNA was subjected to qRT-PCR using the forward and reverse primers of *mir-223*. The results showed significant reduction of *mir-223* gene expression confirming the knockdown of *mir-223* in the extracted RNA sample. This RNA was then subjected to qRT-PCR using the forward and reverse primers for the 13 target transcription factors. β-actin was used as an internal control. We found that knockdown of *mir-223* showed a significant increase in *lbx1b, irf5, zbtb18, irf8, cebpa, ikzf1 and rorca* gene expression and a significant decrease in *spi1a, spi1b*, *mafba, deaf1, rfx1a*, and *tgif1* transcript levels (Figure 4B).

### Functional evaluation of *ikzf1* mutant

Zebrafish *ikzf1* gene (ENSDARG00000013539.10) and the cDNA (ENSDART00000079724.4) sequence and the corresponding human sequences were retrieved from Ensembl. In humans, the IKZF1 gene consists of eight exons and seven introns, and the gene is located on chromosome 7. The zebrafish *ikzf1* gene also contains eight exons and seven introns and the gene is located on chromosome 13. The predicted translational product of the *ikzf1* gene was compared with the human *IKZF1* (ENST00000331340.8) using MultAlin program. Comparison of the human *IKZF1* and the zebrafish *ikzf1* translational product revealed 65% identities and 79% positives between these two proteins (Figure S5).

**Figure 6:**
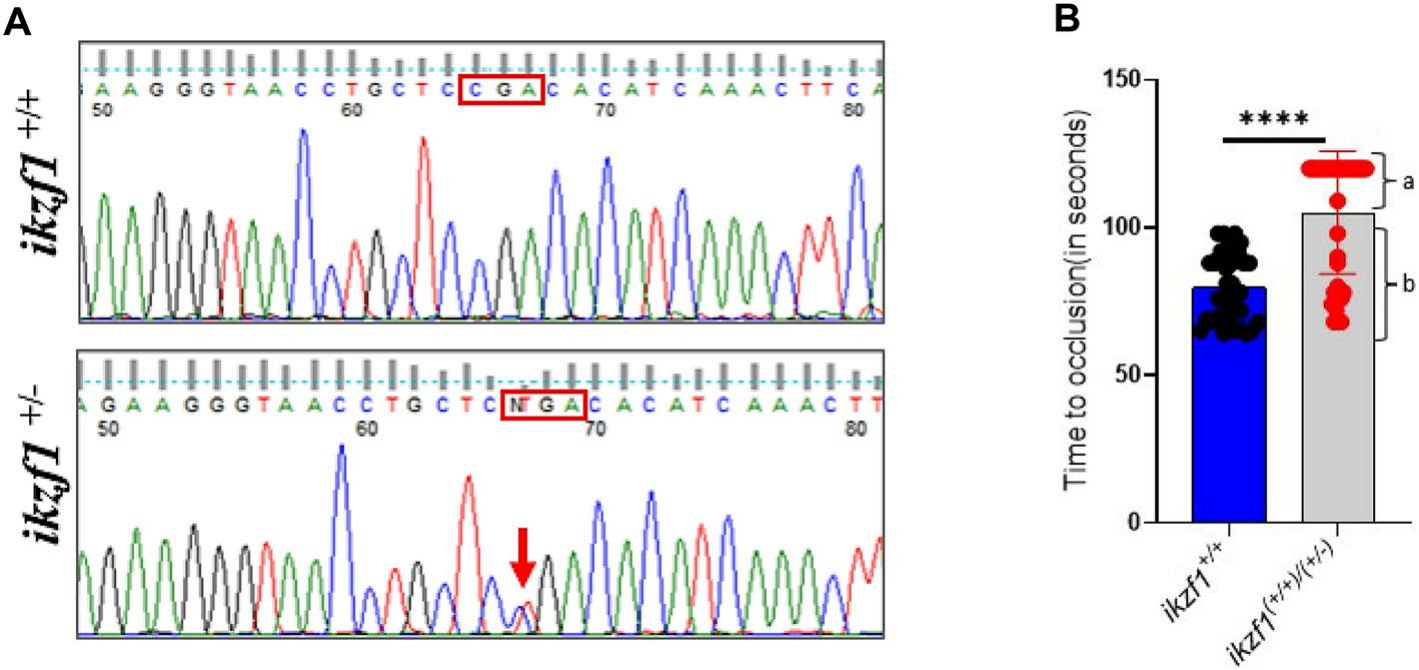
Functional evaluation of *ikzf1* mutant (**A**) Sequence analysis of ikzf1 mutant. The top and bottom sequence chromatograms represent WT *ikzf1*^*+/+*^ and heterozygote *ikzf1*^*+/−*^, respectively. The red boxes in the top and bottom chromatograms show the normal codon with a one peak for C and two peaks C and T, respectively (**B**) Comparison of time to occlusion of the caudal artery after laser injury between 5 dpf WT *ikzf1*^*+/+*^ larvae (blue bars) and a population containing both WT *ikzf1*^*+/+*^ and heterozygote *ikzf1*^*+/−*^ larvae (grey bars). Forty larvae were used in each group experiments (N = 40). The lines on the top represent a significant difference between WT and mutant larvae. Error bars represent mean ± SD. **** represents *p* ≤ 0.0001.

To functionally evaluate the *ikzf1* mutants from ZIRC we grew the mutant embryos to adults and genotyped their DNA isolated from the tail clips. The *ikzf1* exon 5 containing the point mutation was amplified by PCR and was resolved on agarose gel electrophoresis. The gel showed 158 bp amplified band (Figure S6) that was then extracted and sequenced. The sequencing chromatograms showed two peaks, one corresponding to the CGA codon and another with a single nucleotide change in the CGA codon with a substitution of C with T, a point mutation resulting in TGA (Figure 6A). In the cDNA, TGA was at position 531 corresponding to its transcript (ENSDART00000079724.4) leading to a premature stop codon (UGA) and thus at the Arg-168, the protein is terminated. We selected these heterozygotes based on the presence of two peaks and separated them from the WT fish. We obtained 8 females and 1 male. We set up crosses with the only male we obtained with each of these 8 females. The progeny never survived probably due to embryonic lethality. Therefore, the heterozygote *ikzf1* mutant females *ikzf1*^+/−^ were crossed with wild type *ikzf1*^*+/+*^ males from the same progeny and the resulting 5 dpf larvae were subjected to the arterial laser thrombosis and the TTO was recorded. We used 40 larvae in this experiment. Interestingly, the progeny showed two separate populations, group a consisting of 26 larvae that showed TTO >120 seconds, and group b containing 14 larvae that showed an average TTO of 75 seconds (Figure 6B). Thus, the observed ratio of prolonged TTO to normal TTO larvae was 65%:35%. We also quantified thrombocyte numbers in female heterozygotes to ascertain whether there was a reduction in thrombocyte counts among the heterozygous individuals. Our observations reveal a notable decrease in thrombocyte counts compared to wild-type controls originating from the same progeny (Figure S7).

## Discussion

Earlier studies have shown that miRNAs can be inhibited by antagomirs^25,26^ that are chemically modified antisense oligonucleotides that directly bind to the target microRNA preventing it to bind to mRNA that gets degraded by RISC complex^27–29^. Antagomirs were also introduced as a therapeutic approach to downregulate and inhibit abnormally over expressed miRNAs^20^. MicroRNA sponges are engineered from transgenes that can be delivered via viral vectors within the cells to sequester microRNAs because these sponges contain multiple antisense high-affinity binding sites for the miRNA of interest^30,31^. In this paper, we used a cost-effective VMO piggyback knockdown method^32^ to specifically perform knockdowns of microRNAs that are expressed in the zebrafish thrombocytes and tested their effect on thrombocyte production. Our method differs from the other methods in the mechanism of degradation of the miRNA because, it utilizes antisense deoxyoligonucleotides and therefore RNase H is involved in digesting the RNA portion of the RNA-DNA hybrid. Moreover, our target site is not mature miRNA but the microRNA precursor. From our qRT-PCR data, it appears the knockdown is efficient.

In this work, we chose flow cytometry for thrombocyte analysis because it is easy to separate thrombocytes from other blood cells based on their size and because zebrafish thrombocytes are the smallest cells in size compared to other blood cells. Thus, we were able to use flow cytometry to screen the effect of 15 miRNA knockdowns on thrombocyte production. Our finding that *mir-223, mir-7148* and *let-7b* knockdowns increased thrombocyte production suggests that *mir-223, mir-7148* and *let-7b* are repressors that control thrombocyte production.

It has been shown that the low levels of let-7b in neonatal megakaryocytes results in up regulating let-7b target, frizzled family receptor 4 in the WNT regulating pathway and increases mitochondrial biogenesis which drives the neonatal megakaryocyte progenitor cells to maturation and differentiation^33,34^. Our finding that *let-7b* is involved in thrombocyte production is consistent with the findings in megakaryocytes. Likewise, expression profile of miR-223 in cord blood CD34+ cells showed an increase in its expression during megakaryocyte differentiation^35^ through the effects on its target protein LMO2. Over expression of miR-223 in K562 cells showed that it downregulates its LMO2 target protein resulting in an increased megakaryocyte differentiation^36^. These data are consistent with our findings that *mir-223* is important for thrombocyte production and differentiation.

Interestingly, several microRNAs including miR-223 have been shown to play a role in platelet function including agglutination, adhesion and granule secretion^37^. Dysregulation in the expression level of microRNA present in platelets has been associated with thrombin stimulation thus increasing thrombosis episodes^38,39^. In contrast, in our thrombocyte aggregation/agglutination studies, *mir-223* knockdown in adult zebrafish did not show the effect on thrombocyte function. However, in our *mir-223* larval knockdowns, the TTO was shortened in one group of larvae. It is possible that this reduction of TTO is due to increased number of thrombocytes as observed in adult zebrafish and not due to thrombocyte activation. Interestingly, it also showed two other groups, one like the control and the other with prolonged TTO. The larvae that showed TTO similar to the controls are probably the ones where injections were not successful. However, the prolonged TTO group seems paradoxical. We believe that this is due to possible sequestration of thrombocytes that could form microaggregates in larval circulation due to initial thrombocytosis that could lead to prolonged TTO. Nevertheless, the fact that we observed a group with shortened TTO suggests that thrombocytosis also occurs in *mir-223* knockdown larvae. The lack of *mir-223*’s role in thrombocyte function could be due to species-specific variation because miR-223 knockout mice did not show any effect on platelet function, however, in humans it seems it is involved in platelet function[^37^, ^40^, ^41^]. Like *mir-223, let-7b* and *mir-7148* also did not affect thrombocyte function.

*mir-7148* is zebrafish specific microRNA and at present nothing is known about its function in thrombocyte production. This is the first study that showed *mir-7148* is expressed in thrombocytes and its knockdown increased thrombocytes. Also, the TTO results were similar to the *mir-223* findings. In *let-7b* knockdowns, we did not observe three groups of TTO. This is possibly because the thrombocytosis may not be as severe as those observed in *mir-223* and *mir-7148* knockdowns. However, the two groups corresponded to WT and thrombotic phenotypes. Despite these variations all three microRNA knockdowns appear to result in shortened TTO confirming the adult knockdown experiments.

In our experiments we determined thrombocyte counts and performed qRT-PCR on the liver and spleen of the same fish. Since liver and spleen contain a limited number of cell types and loaded with thrombocytes, the efficiency of qRT-PCR may not be affected to a greater extent. The arterial thrombosis experiments in larvae mainly detect thrombocytes in contrast to venous thrombosis that is rich in red cells. If more thrombocytes are present it would aggregate more and cause occlusion more rapidly in arterial thrombosis. This is exactly what happened in our knockdown experiments suggesting more thrombocyte production^42,43^.

In this paper, we found 6 genes, *irf5, tgif1, rfx1a, lbx1b, zbtb18, and deaf1* that showed a significant increase in their expression, 7 genes, *irf8, cebpa, ikzf1, rorca, mafba, spi1a,* and *spi1b* that showed reduction in their expression, and *fhl3b* showed no change in the expression after *let-7b* knockdown. However, *mir-223* knockdown resulted in 7 genes, *irf5, lbx1b, zbtb18,irf8, cebpa, ikzf1, and rorca* that showed a significant increase in their expression, and 6 genes, *tgif1, rfx1a, deaf1 mafba, spi1a, and spi1b* that showed reduction in their expression. Among these the knockdowns *irf5, tgif1, irf8, tgif1, cebpa,* and *rorca* resulted in thrombocytosis whereas *ikzf1* knockdown resulted in thrombocytopenia. Thus, 5 genes that showed reduction in expression following *let-7b* and *mir-223* knockdowns that yielded thrombocytosis are *tgif1, irf8, tgif1, cebpa,* and *rorca* and one gene, *ikzf1* resulted in reduction in expression following *let-7b* knockdown that yielded thrombocytopenia. Since overall, the *let-7b* and *mir-223* knockdowns led to thrombocytosis, it appears the result is due to the cumulative action of the downstream targets; in some cases, they could be repressors and, in some other cases, they could be activators. Moreover, these two microRNAs can independently increase or decrease these target transcripts. For example, knockdown of *let-7b* resulted in decrease of *ikzf1* whereas *mir-223* knockdown resulted in increase in *ikzf1* transcripts. Thus, the cumulative effect of these microRNAs is to keep the balance of the total *ikzf1* transcripts which results in enhanced thrombopoiesis. Another result is that *irf5* increases after *let-7b* and *mir-223* knockdowns but a reduction in *irf5* shows a group of larvae with shortened TTO that is consistent with thrombocytosis, and this is contradicting. This is perhaps because the regulation is cumulative effect of the target factors on thrombopoiesis and is not an isolated event controlled by *irf5*. Recent studies have shown that a few microRNAs upregulate transcription of the target genes as well as the translation of target mRNA^44,45^. Studies on the above genes have shown their role in different aspects of hematopoiesis. For example, in mice, knockout studies of IRF8 and TGIF have shown that these factors play a role in upregulation of immature myeloid cell production and myeloid progenitor cell differentiation, respectively^46–48^. RORCA and CEPBA have been shown to be involved in regulating thymopoiesis^49,50^ and myeloid cell differentiation, respectively^51–53^. In a rheumatoid arthritis patient miR-223 was upregulated whereas RORCA was downregulated in comparison with healthy control^54^. This is contrariwise to our observation that *mir-223* knockdown upregulated the Rorca expression in zebrafish. However, none of these studies has shown that these factors control thrombopoiesis and our current finding suggests that they are involved in thrombopoiesis and are under the control of microRNAs.

Our results showed *ikzf1* knockdown resulted in thrombocytopenia suggesting Ikzf1 is an activator for thrombopoiesis. These results are also supported by our studies on *ikzf1* mutants which showed a dominant, prolonged TTO phenotype because the progeny of heterozygote and wild-type yielded a mixture of two populations, one with the WT TTO and the other with the prolonged TTO. Interestingly, individuals with IKZF1 haploinsufficiency manifest immune thrombocytopenia (ITP)^55^. Our results of *ikzf1* heterozygote adults showing thrombocytopenia are consistent with the above findings. Lenalidomide degrades IKZF1 and releases its inhibition of megakaryocytic gene promotor such that GATA2 transcription factor complex binds promoter to induce megakaryocyte differentiation and is similar to the IKZF1 knockout results in mice^56–58^. However, in another study in contrast to the above finding, degradation of IKZF1 results in down-regulation of its target GATA1 followed by the downregulation of NFE2 resulting in thrombocytopenia^57,59^. Our results on *ikzf1* are not only consistent with the GATA1 results that led to thrombocytopenia but also matches with the haploinsufficiency of IKZF1 in humans.

Our laser-induced arterial thrombosis following the knockdown of *irf8* showed a shortened TTO and yielded thrombotic phenotype. However, *ikzf1* knockdown showed a prolonged TTO matching the thrombocytopenic phenotype seen in adult knockdown experiment. These are consistent with thrombocytosis and thrombocytopenia, respectively. Interestingly, *rorca* and *tgif1* knockdowns did not result in shortened TTO but yielded prolonged TTO. Our observation of microthrombi in these knockdowns suggests that clots along with thrombocyte aggregation occurred that lead to the consumption and sequestration of the circulating thrombocytes causing exacerbated thrombocytopenia and hence the observed prolonged TTO. The microthrombi formation could also be due to the effects of factors extraneous to thrombocyte contribution. For example, endothelial contribution may be involved. Likewise, *cebpa* knockdown in larvae yielded prolongation of TTO, however, did not show microthrombi probably because its effect may be due to mild thrombocytopenia. Interestingly, in *irf5* knockdown, a group of larvae had a thrombotic phenotype as noted above, and thus, *irf5* may be similar to *irf8*.

In our investigations, we performed knockdown experiments targeting transcription factors that exhibited reduced expression after *let-7b* knockdown. Nevertheless, further research is needed to examine the effects of overexpressing factors that demonstrated heightened levels upon *let-7b* knockdown. Moreover, a comprehensive understanding of these factors' intricate regulation of thrombocyte production warrants the integration of knockdown and overexpression approaches in tandem and in combination.

Taken together these results suggest that *mir-223, let-7b and mir-7148* are important repressors of thrombocyte production. Our results also showed that *mir-223* and *let-7b* regulate multiple transcription factors both in a positive and negative manner. This information should lead to future understanding of the physiological role of these targets in thrombopoiesis. In conclusion, the inhibition of thrombocyte production by microRNAs is via a complex network of transcription factors downstream of the microRNAs.

### Supplementary Information

Supplementary Information 1.

Supplementary Information 2.

## Data Availability

The datasets generated and/or analysed during the current study are available in the PRJNA753153 repository, (https://www.ncbi.nlm.nih.gov/bioproject/PRJNA753153).
