# Supplementary material for: Role of microRNAs and their downstream target transcription factors in zebrafish thrombopoiesis
Source: Sci Rep. 2023 Sep 26;13:16066. doi: 10.1038/s41598-023-42868-7 (PMC10522587; doi:10.1038/s41598-023-42868-7)
Supplement: Supplementary file 1 — Supplementary Information 1. [file 41598_2023_42868_MOESM1_ESM.pdf]

Supplementary Data

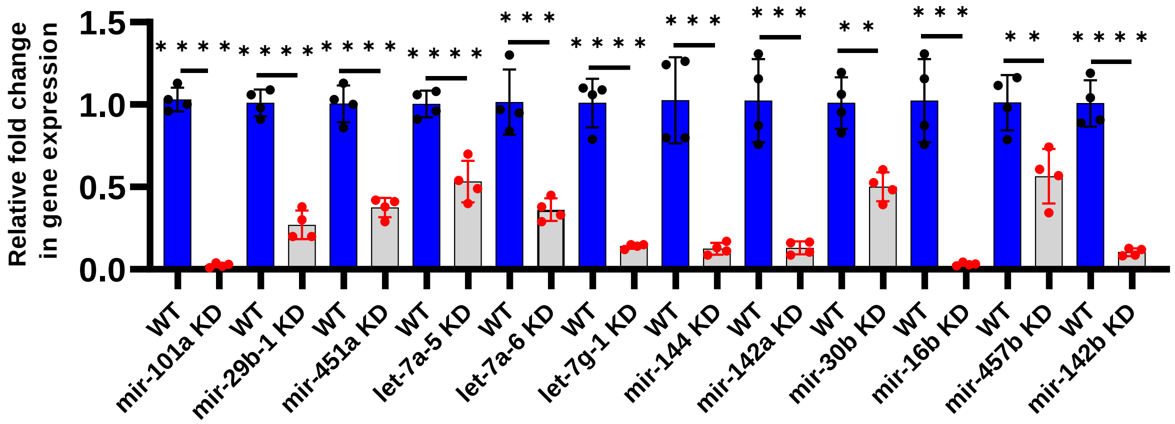

Figure S1. Quantitative real-time PCR showing the fold change of the microRNA expression in daily wild-type (WT) controls (blue bars) and knockdown (KD) samples (grey bars). Four replicates containing six injected fish were used for each replicate sample and control experiments (N = 4). The lines on the top of the bars represent a significant difference between WT and knockdown samples. Error bars represent mean ± SD. *, **, ***, and **** represent $p \leq 0.05$, $p \leq 0.01$, $p \leq 0.001$, and $p \leq 0.0001$, respectively.

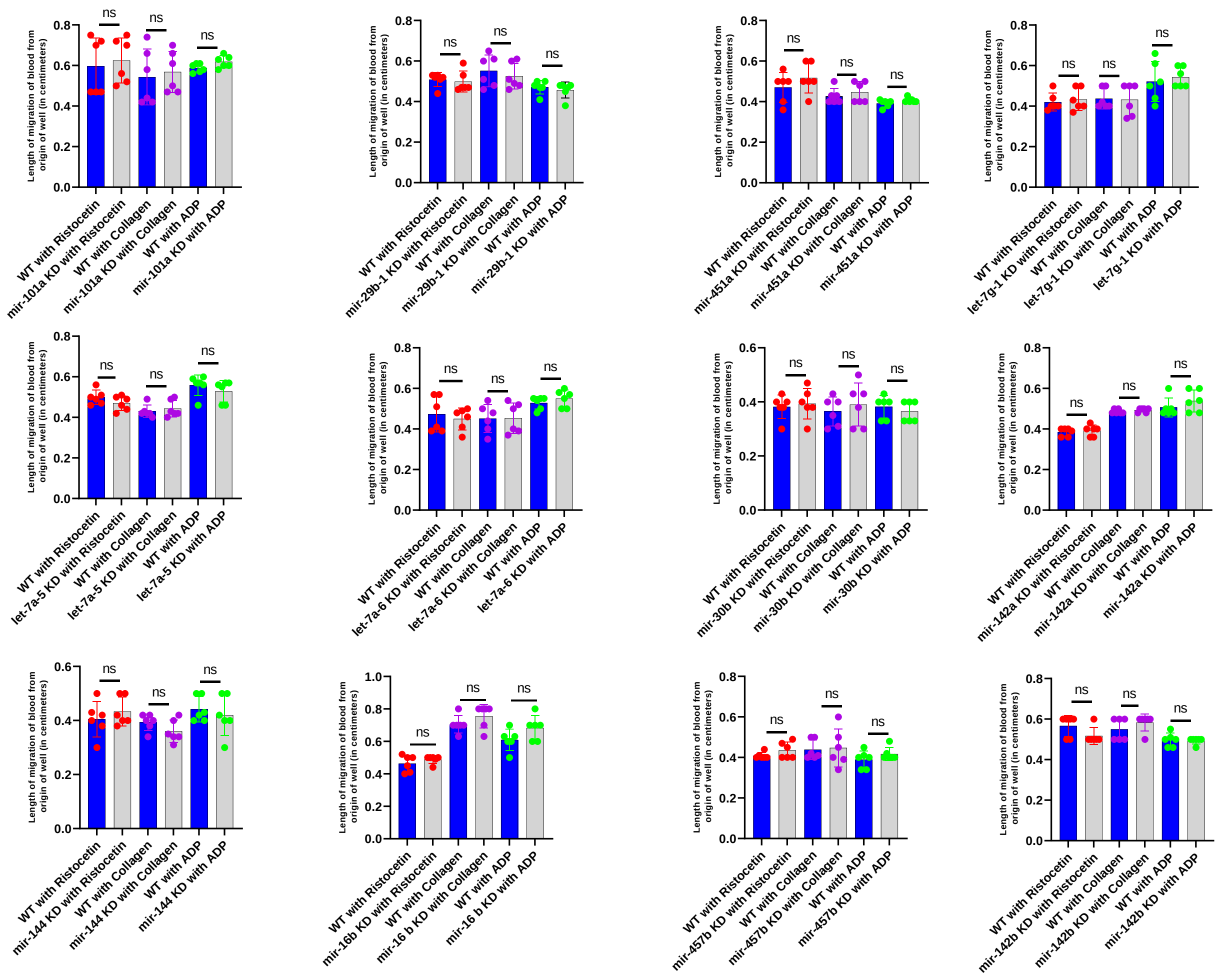

Figure S2. Effect of knockdowns of twelve microRNAs in adult zebrafish on thrombocyte aggregation/agglutination. Zebrafish blood samples from the daily controls (WT, represented as blue bars) and the knockdown (KD), represented as grey bars) samples were subjected to whole blood aggregation/agglutination assay with ristocetin, collagen and ADP. The length of migration of blood from the origin of the well down the wall was measured and plotted. The results between WT and KD samples were compared. Six fish were used for each of the knockdown and control experiments (N = 6). Error bars represent mean ± SD. ns (non-significant) represents p >0.05.

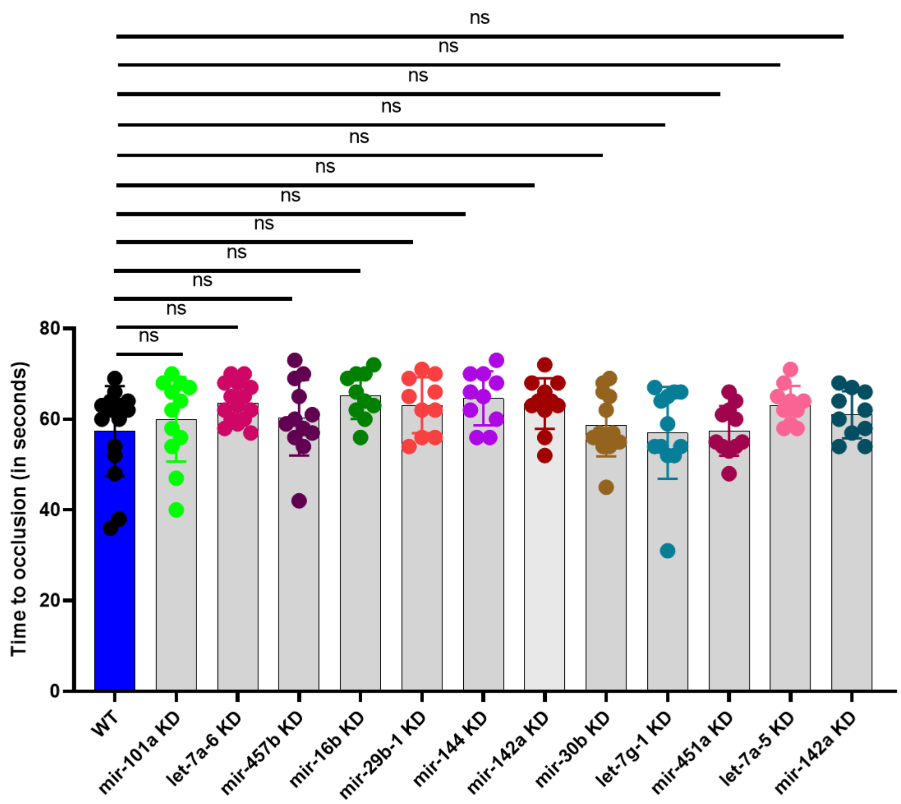

Figure S3. Arterial thrombosis in knockdown larvae of twelve microRNAs. (A) Comparison of time to occlusion of the caudal artery after laser injury between daily WT (blue bar) and knockdown (grey bars) 5 dpf larvae. The number of larvae used were 15 for daily control WT larvae and 10-15 larvae for individual KD microRNAs, respectively. Students' t-test was used for statistical analysis. Error bars represent mean ± SD. p-value > 0.05 was considered non-significant (ns) represented by the lines on the top.

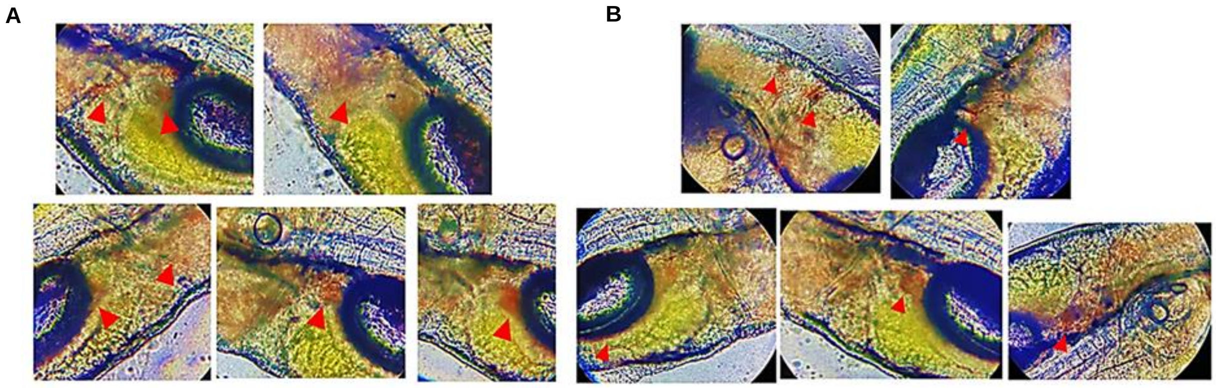

Figure S4. Representative images of 5 dpf zebrafish larvae 48 hours post piggyback knockdown of (A) *rorca* and (B) *tgif1*. Arrowhead shows microthrombi at multiple locations.

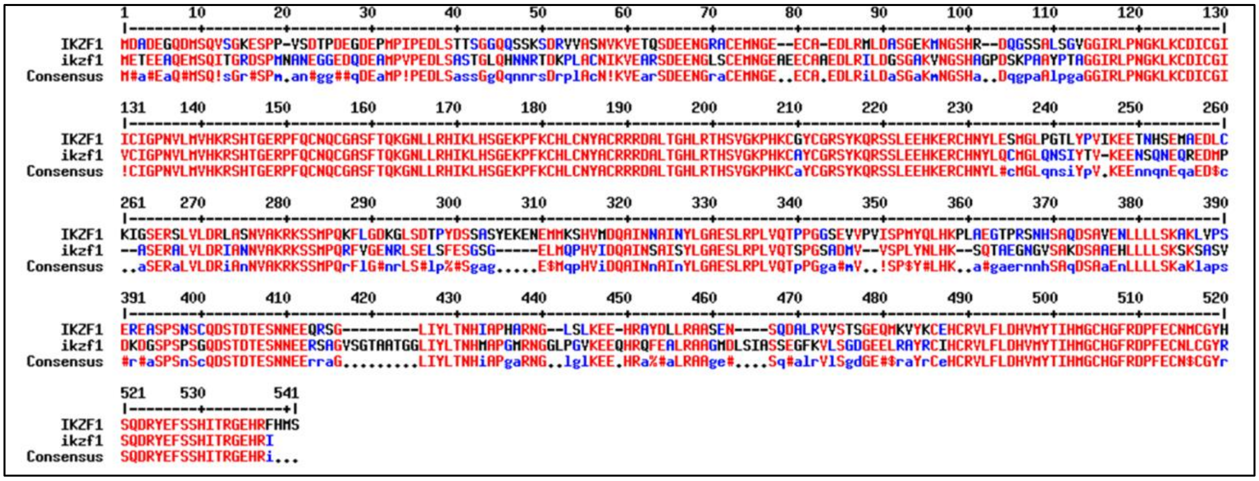

Figure S5. MultAlin comparison of the zebrafish Ikzf1 and human IKZF1 protein sequences. The amino acids letters labeled in red, blue and black are high consensus (conserved), low consensus, and neutral, respectively.

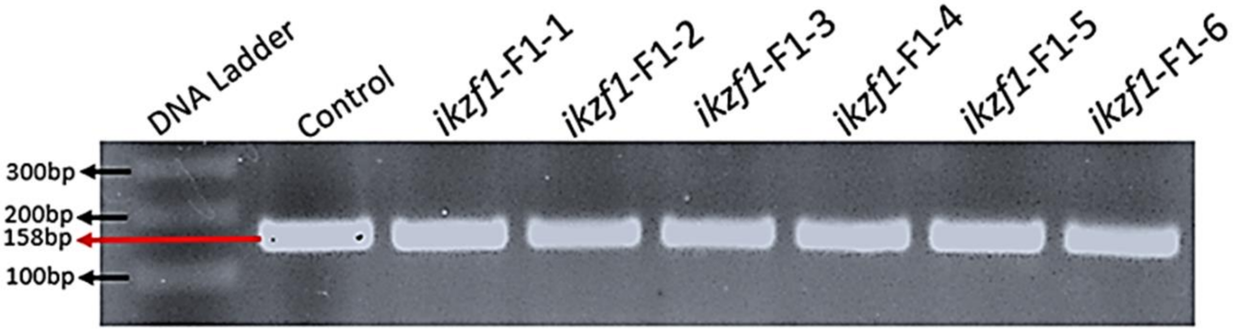

Figure S6. Agarose gel electrophoresis showing representative PCR amplified products of genomic DNA from tail clips of the adults grown from *ikzf1* mutant embryos obtained from ZIRC. The original gel is shown below.

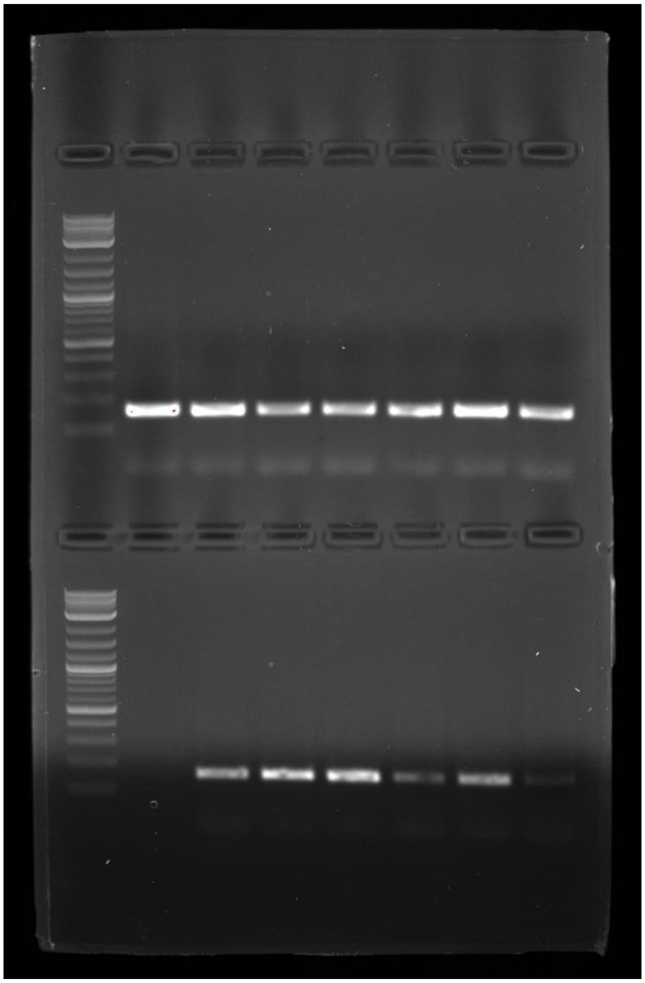

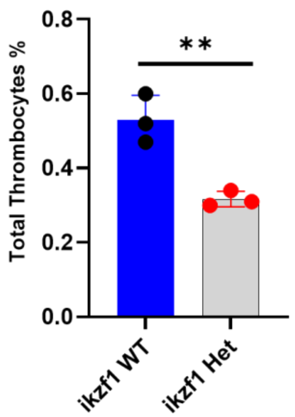

Figure S7. Comparison of the percentage of thrombocytes by flow cytometric analysis in whole blood from WT controls (blue bars), and *ikzf1* hets (grey bars). Three fish were used for control and hets (N = 3). The line on the top of the bars represent a significant difference between WT and knockdown sample sets. Error bars represent mean ± SD. ** represents $p \leq 0.01$. p-value < 0.05 was considered significant.

Table S1. List of the forward (FP) and reverse (RP) primers used in qRT-PCR.

Gene Name	qRT-PCR primers (5′ to 3′)
mir-101a	**FP:** GGCTGCCCTGGTTCAGTTAT
	**RP:** GGCAGCCATCCTTCAGTTAT
mir-29b-1	**FP:** CTGCTCCTGGAAGCTGAATT
	**RP:** CGGCCCAGGAACACTGATTT
mir-451a	**FP:** AGAGGCGGCGAAACCGTTAC
	**RP:** AAAGGCAGCAGAACCCTTAC
let-7a-5	**FP:** GTACGTGTTTTTGGTGTCTG
	**RP:** GCAGACCTTAGGTGACACAA
let-7a-6	**FP:** CACAGTGAACCTGTGTGTTT
	**RP:** CACTCGTGGTCATTGTGTCT
let-7g-1	**FP:** GGGGGCTGTGGAATGAGGTA
	**RP:** GGAGCAGCTGTGAGAAAGAC
mir-144	**FP:** GCTCTCTAGACAGGATATCA
	**RP:** GCTCGTTGACCCCCTGGATA
mir-142a	**FP:** CGTACAGTGCAGTCATCCAT
	**RP:** CAACAGTACACTCATCCATA
mir-30b	**FP:** TTCCAGTGTAGTCGCTGTAA
	**RP:** CTCCAGACAGTCACAGCAAA
mir-16b	**FP:** ACTTGGCCGTGTGACAGACT
	**RP:** CTCCAGCAGCACGGTCAATA
mir-457b	**FP:** GAATGTACTAAAGCAGCACA
	**RP:** GGTCTTACTACAGCAGAACA
mir-142b	**FP:** ACAGTGCAGTCACTCATAAA
	**RP:** ACAGTACACTCATCCATAAA
